# Effect of mothers‘ health literacy on early childhood allergy prevention behaviours: results from the KUNO-Kids health study

**DOI:** 10.1186/s12889-024-19906-8

**Published:** 2024-09-05

**Authors:** Maja Pawellek, Angela Köninger, Michael Melter, Michael Kabesch, Christian Apfelbacher, Susanne Brandstetter, KUNO-Kids study group, Andreas Ambrosch, Petra A. Arndt, Andrea Baessler, Mark Berneburg, Stephan Böse-O’Reilly, Romuald Brunner, Sara Fill Malfertheiner, André Franke, Robert Häsler, Sebastian Häusler, Iris Heid, Stefanie Heinze, Wolfgang Högler, Sebastian Kerzel, Michael Koller, Michael Leitzmann, Áine Lennon, David Rothfuß, Wolfgang Rösch, Bianca Schaub, Stephan Weidinger, Sven Wellmann

**Affiliations:** 1 University Children’s Hospital Regensburg (KUNO), Regensburg, Germany; 2 Member of the Research and Development Campus Regensburg (WECARE), Hospital St. Hedwig of the Order of St. John, Regensburg, Germany; 3 University Clinic of Gynecology and Obstetrics, Hospital St. Hedwig of the Order of St. John, Regensburg, Germany; 4 Institute of Social Medicine and Health Systems Research, Otto Von Guericke University Magdeburg, Magdeburg, Germany

**Keywords:** Health literacy, Mothers, Early childhood allergy prevention, Health behaviour, Child, Cohort study

## Abstract

**Background:**

Parents can engage in several behaviours with regard to early childhood allergy prevention (ECAP). These can be related to diet of mother/child and the modification of the home environment; not all of them are justified by current evidence. Previous studies showed that parental health literacy (HL) is related to favourable health behaviours directed at the child. This study aimed to investigate the causal effect of mothers’ HL on ECAP behaviours and to test different moderators of this effect.

**Methods:**

One thousand six hundred sixty-two mothers participating in the KUNO-Kids health study in the area of Regensburg, Germany were surveyed on HL (assessed via the health care scale of the Health Literacy Survey-EU questionnaire, HLS-EU-Q47) and ECAP behaviours implemented during pregnancy and the child’s first year of life. Patterns in ECAP behaviours were identified by latent class analysis. Multinomial regression modelling was performed with HL as exposure, ECAP as outcome variable, allergy risk, parental competence and bonding, anxiety and depression as moderators as well as potentially confounding variables.

**Results:**

We identified three classes of ECAP behaviours (class 1: „breastfeeding “ *N* = 871; class 2: „allergen-avoidance “ *N* = 490; class 3: „mixed behaviours “ *N* = 301). In univariable as well as fully adjusted regression models, compared to class 1, class 2 was negatively, and class 3 was not associated with HL. None of the tested moderating variables altered the association between HL and ECAP significantly.

**Conclusions:**

We found an effect of mothers’ HL on ECAP behaviours: lower HL of mothers increased allergen-avoiding behaviour directed at their child, while decreasing the chance of exclusive breastfeeding. Improving HL could contribute to the implementation of recommended ECAP behaviours in families, especially to the reduction of allergen-avoiding behaviours.

**Supplementary Information:**

The online version contains supplementary material available at 10.1186/s12889-024-19906-8.

## Background

Health literacy (HL) describes the knowledge, motivation and competencies of accessing, understanding, appraising and applying health-related information in healthcare, disease prevention and health promotion [1]. Recent studies showed associations of high parental HL and various positive health behaviours directed at the child and favourable child health outcomes [2–4]. However, most studies assumed direct associations between HL and health behaviours and tested simple models or models that included confounding variables like sociodemographic, psychosocial or cultural factors [4]. Only few studies considered more complex relationships between HL and health behaviours and tested more elaborated models including additional variables like mediators or moderators [5–8]. However, unlike mediators such as self-efficacy or attitudes and beliefs, only few studies included potential moderators in their analyses like education or residency. These moderators have not been proven to significantly moderate the effect of HL on health behaviours [6, 9, 10]. Overall, the current understanding of how and when HL translates into health behaviours is still limited, partly due to insufficient modelling strategies.

Early childhood allergy prevention (ECAP) is an umbrella term for parental behaviours with regard to children’s exposure to or avoidance of allergens in the field of nutrition or living environment. Despite earlier recommendations to avoid potential allergens, the prevalence of allergies remained high in the 2000s, so that more and more studies investigated the effect of early introduction of solid foods on the development of allergies. Based on an initial descriptive study of the prevalence of peanut allergy in the UK and Israel [11], the LEAP study started in 2006 with a randomized controlled trial. They found that early introduction of peanuts significantly decreased the incidence of peanut allergy among high-risk children [12]. Subsequently, a paradigm shift in allergy prevention from avoidance to exposure has occurred and has questioned many measures formerly presumed efficacious. Changes in guidelines on allergy prevention followed; in Germany, revised guidelines were published in 2014 and 2022 [13, 14]. Contrary to previous recommendations, recent research suggests that early exposure to potential allergens (such as peanuts, eggs, and milk) to infants may be beneficial [15]. Concerning allergen avoidance in the home environment (e.g., minimizing exposure to house dust mites) the effectiveness varies and recent guidelines even discourage measures for reducing house dust mites [13, 16, 17]. However, regardless of justification by current evidence, parents implement several behaviours with the aim of primary prevention of different allergic diseases, such as asthma, allergic rhinitis, food allergy and atopic eczema. In a previous study we investigated whether and to what extent families in Germany engage in ECAP behaviours. We found that most parents practice behaviours like early introduction of solid foods, exclusively breastfeeding for at least four months or not exposing the child towards smoke, but allergen-avoidance measures (like avoidance of specific foods or measures against house dust mites) were also implemented – in both children at-risk and not at-risk for allergies [18].

While the effect of parental HL on specific behaviours which are part of allergy prevention has been studied (such as breastfeeding [19] or smoke exposure [20, 21]), until now, to the best of our knowledge a comprehensive set of different behaviours in the field of nutrition and living environment has not yet been addressed by research. Therefore, the objective of this study was to investigate the causal effect of HL on ECAP behaviours in a large cohort of new mothers in Germany. For this purpose the following research questions were pursued:

- Which patterns of ECAP behaviours are practiced in German families?
- Is there an effect of mothers’ HL on ECAP behaviours?
- Is the effect of HL on ECAP behaviours moderated by third variables?

## Methods

### Design

This observational study used data from the KUNO-Kids health study, a prospective birth cohort study which is conducted in the hospital St. Hedwig in Regensburg (East Bavaria, Germany) [22]. The study has been approved by the Ethics Committee of the University of Regensburg (reference numbers: 14–101-0347, 19–1646-101).

### Participants and data collection

Women were approached in the last trimester of pregnancy or during their hospital stay after delivery and were invited to participate in the study. Mothers were eligible for enrolment if they were at least 18 years old and if they were able to provide informed consent. Newborns (and their families) were eligible for participation in the KUNO-Kids health study if no older sibling had already been included. Data considered in this manuscript was collected at four time points: directly after birth (baseline), after four weeks, after six months and after one year. At baseline, data was collected using a standardized computer-assisted personal interview and paper-based self-report questionnaires. For follow-up measurements, paper-based self-report questionnaires were sent to the families. Recruitment of the KUNO-Kids study started in June 2015. In the analysis sample we included participants who were recruited until March 2020 and who participated for at least one year in the study (with the last assessment in March 2021).

### Measures

#### Outcome: ECAP

ECAP behaviours were assessed at all four time points in order to assess them as closely over time as possible. They comprised behaviours related to the diet of mother and child and the home environment. We applied latent class analysis (LCA) to identify different classes of ECAP behaviours. Variables were included in LCA if they had a minimum frequency of 10% for each response category and if they were not conditioned on another variable: fish as part of mothers’ diet during pregnancy (≥ 1 per week/ < 1 per week); duration of exclusively breastfeeding (no breastfeeding/ < 4 months/ ≥ 4 months); regular feeding of hydrolysed infant formula (hydrolysed formula/other formula); child’s age of introduction of any solid foods (< 4 months/4–6 months/ > 6 months); allergy prevention related avoidance of feeding specific foods in the child’s diet during the first year of life (dairies, wheat, hen egg, fish, meat, nuts (incl. peanuts), soy, citruses, other fruit or vegetable, other foods): yes (any)/no; fish as part of solid foods during the child’s first year of life (≥ 1 per week/ < 1 per week); feeding of farm milk (yes (cow milk not boiled/boiled/goat milk)/no); measures for reducing house dust mites (removal of carpets, additional cleaning, use of specific vacuum cleaners, mattress encasing, allergy mattress, allergy pillow/blanket): yes (any)/no; and exposure to tobacco smoke by smoking of parents or in the child’s home: yes/no. Contact to hay, smoking during pregnancy and avoidance of pets were not considered in LCA since prevalence was too low (< 10%).

#### Exposure variable: HL

Mothers’ HL was assessed at baseline with the German version of the health care scale of the standardized HLS-EU-Q47 questionnaire [23] (internal consistency: Cronbach’s alpha = 0.91 [24]). It consists of 16 items that are scored on a 4-point Likert scale (“very difficult – quite difficult – quite easy – very easy”). For each person a mean score was calculated and transformed to a metric from 0 to 50 to be able to compare our results with other studies using the HLS-EU questionnaire [25]. In case of item-level missing values multiple imputation by chained equations methods were applied.

#### Confounding and moderating variables

**Fig. 1:**
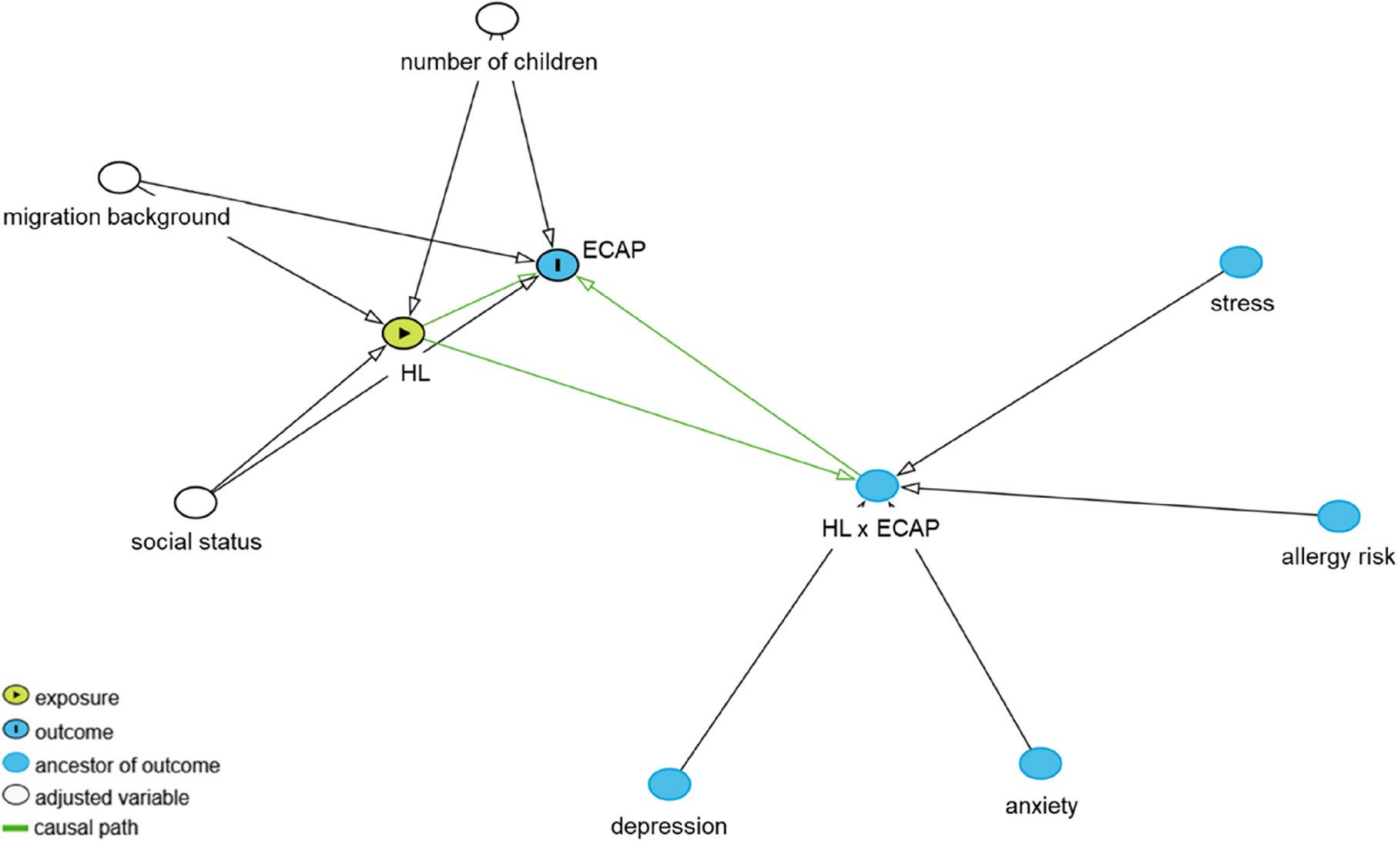
Directed acyclic graph representing the independent effect of exposure HL on outcome ECAP, adjusted for number of children, migration background and social status, and modification of this effect by stress, allergy risk, anxiety and depression. The graph was drawn using the online application DAGitty (https://www.dagitty.net/)

Variables possibly acting as moderators of the HL-ECAP effect and a set of confounders were identified based on empirical findings summarized in a recent review of the literature [4]. Then, availability of variables in the KUNO-Kids study was checked and the resulting analytical models were specified based on directed acyclic graphs methodology (DAG, [26], see Fig. 1). In order to avoid multicollinearity only the most prominent variables of relevant topics (e.g. health variables) were selected based on prior theoretical consideration.

Variables that might act as moderators on the effect of mothers’ HL on ECAP comprised: allergy risk status (mother, father and/or sibling having an allergic disease (food allergy, allergic rhinitis, allergic conjunctivitis, bronchial asthma, atopic dermatitis): yes/no); EBI (“Eltern-Belastungs-Inventar”, [parenting stress index]) parental competence scale [27]; EBI parental bond scale [27]; PHQ-D (Patient Health Questionnaire) depression scale (PHQ-9: 0–4 = no depression, 5–9 = mild depression, 10–27 = major depression) [28], PHQ-D anxiety scale (GAD-7: question “How often have you felt affected by the following complaints in the last 4 weeks? Nervousness, anxiety, tension or excessive worry” and three or more of the following questions are answered with "on more than half of the days" = yes, else = no) [28].

Variables considered as potential confounders of the effect of mothers’ HL on ECAP comprised: migration background (country of birth Germany/other); subjective social status [29]; number of children (one child/more children).

An English language version of the items considered for this analysis can be found in Supplementary file I.

### Statistical analysis

Descriptive data is presented as mean (standard deviation, SD), categorical data is presented in frequencies (%). Results of the regression analyses are presented as odds ratio (OR) with 95% confidence intervals. Selection bias was investigated by comparing general characteristics (age, education, etc.) at baseline between participants who were and were not included in the analysis sample, respectively (see Participants and data collection), using t-test, Mann–Whitney-U test and chi-square test.

The total amount of missingness throughout the dataset was 10.2%. To account for missing values multiple imputation by chained equations methods were performed using the MICE package in R [30]. Results of ten multiple imputations were combined by computing the mean or selecting the most likely imputed value and analyses were performed on this merged data set. We additionally checked for patterns and randomness of missing data.

LCA was performed to identify patterns of ECAP behaviours. LCA models identify latent classes within which observed categorical indicators follow the same distribution and probabilities for class membership are generated [31]. We fitted latent class models with one to six classes to determine the optimal number of latent classes (see Supplementary file II). The best fitting model was identified using Bayesian information criteria (BIC), Lo-Mendel-Rubin-Test (LMR) and the size of the smallest class [31].

In order to analyse the effect of mothers’ HL on ECAP, multinomial regression models were performed as we used ECAP as a variable with three distinct classes without ordered relationship. Univariable (model 0) and multivariable regression models were fitted, including different moderators as multiplicative interaction terms in the regression models (model 2: allergy risk status; model 3: parental competence; model 4: parental bond; model 5: depression; model 6: anxiety). All multivariable models were adjusted for migration background, subjective social status and number of children.

Statistical analyses were performed according to an a priori specified analysis plan [32]. The reporting follows the guidelines for reporting observational studies (STROBE statement, [33], see STROBE checklist Supplementary file III). Data were cleaned using SPSS version 28 (IBM Corp. Released 2021. IBM SPSS Statistics for Windows, Version 28.0.0.0 Armonk, NY: IBM Corp). All analyses were conducted using R version 4.1.1 (2021–08-10; The R Foundation for Statistical Computing; Vienna, Austria), LCA was performed using the R package “poLCA” [34].

## Results

### Descriptive analyses

The total sample of mothers recruited for the KUNO-Kids health study until March 2020 comprised *N* = 3199. 1537 mothers were excluded due to drop-outs before 1-year assessment, *N* = 1662 mothers were included for analyses. The characteristics of the total sample and the analysis sample are displayed in Supplementary file IV. Compared to the drop-out sample mothers from the analysis sample were more often educated at university entrance level, married and living together with their partner, employed before pregnancy, and had less often migration background (all *p* < 0.001).

In Table 1 the baseline characteristics for included participants are presented. Mothers’ mean HL score at baseline was 35.83 (*SD* = 7.3) (Cronbach’s alpha at baseline: 0.90), more than a third (36.5%) showed limited HL (score range 0 – 32; categorization according to [35]).

**Table 1:** Baseline characteristics of participants (*N* = 1662)

Characteristic	N	N (%)	Mean (SD)
***Sociodemographic characteristics***			
Age (years)	1647		32.61 (4.10)
Marital status	1629		
Married, living together with husband		1333 (81.83%)	
Unmarried, living together with partner		271 (16.64%)	
Living without partner/ divorced/ widowed		25 (1.53%)	
Maternal education	1626		
No degree or less than 10 years of schooling		107 (6.58%)	
Ten years of schooling		494 (30.38%)	
University entrance level		1025 (63.04%)	
Maternal employment before pregnancy	1627	1481 (91.03%)	
***Exposure variable***			
HL (0–50)	1560		35.83 (7.42)
***Confounding and moderating variables***			
Allergy risk status	1499	848 (56.57%)	
EBI parental competence scale (4–20)	1489		7.76 (3.72)
EBI parental bond scale (4–20)	1483		8.55 (3.38)
PHQ-D depression scale	1432		
No depression		878 (61.31%)	
Mild depression		434 (30.31%)	
Major depression		120 (8.38%)	
No anxiety (PHQ-D anxiety scale)	1472	1443 (98.03%)	
Migration background (country of birth Germany)	1631	1482 (90.86%)	
Subjective social status (0–10)	1473		6.75 (1.25)
Primiparous	1644	1048 (63.75%)	

*SD* Standard deviation, *HL* Health literacy score (0–50), *EBI* “Eltern-Belastungs-Inventar” (parenting stress index), *PHQ-D* Patient Health Questionnaire

### Latent class analysis

**Fig. 2:**
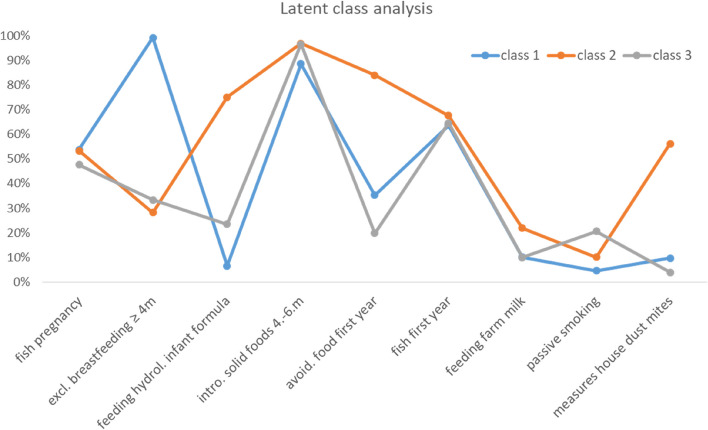
Results of latent class analysis: class 1 “breastfeeding” (*N* = 871), class 2 “allergen-avoidance” (*N* = 490), class 3 “mixed behaviours” (*N* = 301). Variables included in LCA: fish as part of mothers’ diet during pregnancy (≥ 1 per week vs. < 1 per week); duration of exclusively breastfeeding (≥ 4 months vs. no breastfeeding/ < 4 months); regular feeding of hydrolysed infant formula (hydrolysed formula vs. other formula); child’s age of introduction of any solid foods (4–6 months vs. < 4 months/ > 6 months); allergy prevention related avoidance of feeding specific foods in the child’s diet during the first year of life (yes vs. no); fish as part of solid foods during the child’s first year of life (≥ 1 per week vs. < 1 per week); feeding of farm milk (yes vs. no); exposure to tobacco smoke (yes vs. no); measures for reducing house dust mites (yes (any) vs. no).

According to model fit indices a model with three classes showed the best fit (BIC = 16,967.8; LMR: LR(13) = 241.78, *p* < 0.001; smallest class size: 18.57%). Class 1 “breastfeeding” (*N* = 871) was mostly characterized by exclusively breastfeeding for at least four months, class 2 “allergen-avoidance” (*N* = 490) by a lower probability for breastfeeding and more pronounced avoidance of allergens and class 3 “mixed behaviours” (*N* = 301) by a similar pattern to class 1 but a lower probability of exclusively breastfeeding. Results are displayed in Fig. 2.

### Regression analyses

In the univariable model (model 0), compared to class “breastfeeding”, class “allergen-avoidance” was negatively (OR = 0.968, *p* < 0.001), and class “mixed behaviours” was not associated (OR = 0.999, *p* = 0.905) with mothers’ HL. After adjustment for confounders (model 1) class “allergen-avoidance” was still negatively (OR = 0.971, *p* < 0.001) and class “mixed behaviours” not associated (OR = 1.00, *p* = 0.603) with HL.

Models 2 to 6 included each a moderator variable, however, in none of these models the association between mothers’ HL and ECAP behaviours was significantly altered by the moderator variable (allergy risk status, parental competence, parental bond, depression or anxiety). Trends were visible for parental bond (class “mixed behaviours”: OR = 0.995, *p* = 0.085) and mild depression (class “allergen-avoidance”: OR = 0.968, *p* = 0.072). Results are presented in Table 2.

**Table 2:** Regression models for the association between HL and ECAP (class “breastfeeding” as reference) (*N* = 1662)

		**Model 0**		**Model 1**		**Model 2**		**Model 3**		**Model 4**		**Model 5**		**Model 6**	
**Exposure/ moderator**	**Outcome**	**Estimate (SE)**	**OR (95% CI)**	**Estimate (SE)**	**OR (95% CI)**	**Estimate (SE)**	**OR (95% CI)**	**Estimate (SE)**	**OR (95% CI)**	**Estimate (SE)**	**OR (95% CI)**	**Estimate (SE)**	**OR (95% CI)**	**Estimate (SE)**	**OR (95% CI)**
**HL**	**ECAP class “allergen-avoidance”**	**-0.03 (0.01)**	**0.97 (0.95–0.98)**	**-0.03 (0.01)**	**0.97 (0.96–0.99)**	**-0.03 (0.01)**	**0.97 (0.95–1.00)**	-0.03 (0.02)	0.97 (0.93–1.01)	0.01 (0.02)	0.99 (0.95–1.04)	-0.02 (0.01)	0.98 (0.96–1.00)	**-0.03 (0.01)**	**0.97 (0.96–0.99)**
	**ECAP class “mixed behaviours”**	0.00 (0.01)	1.00 (0.98–1.02)	0.01 (0.01)	1.00 (0.99–1.02)	-0.01 (0.02)	0.99 (0.95–1.02)	0.03 (0.02)	1.03 (0.99–1.08)	0.05 (0.03)	1.05 (1.00–1.10)	0.02 (0.01)	1.02 (0.99–1.04)	0.01 (0.01)	1.01 (0.99–1.02)
**Allergy risk**	**ECAP class “allergen-avoidance”**	-	-	-	-	-0.28 (0.58)	0.76 (0.24–2.33)	-	-	-	-	-	-	-	-
	**ECAP class “mixed behaviours”**	-	-	-	-	-0.55 (0.74)	0.58 (0.14–2.45)	-	-	-	-	-	-	-	-
HL*allergy risk	**ECAP class “allergen-avoidance”**	-	-	-	-	-0.01 (0.02)	0.99 (0.96–1.03)	-	-	-	-	-	-	-	-
	**ECAP class “mixed behaviours”**	-	-	-	-	0.03 (0.02)	1.03 (0.99–1.07)	-	-	-	-	-	-	-	-
**EBI parental competence**	**ECAP class “allergen-avoidance”**	-	-	-	-	-	-	-0.01 (0.08)	0.99 (0.85–1.16)	-	-	-	-	-	-
	**ECAP class “mixed behaviours”**	-	-	-	-	-	-	0.15 (0.09)	1.16 (0.97–1.38)	-	-	-	-	-	-
HL*EBI parental competence	**ECAP class “allergen-avoidance”**	-	-	-	-	-	-	0.00 (0.00)	1.00 (1.00–1.00)	-	-	-	-	-	-
	**ECAP class “mixed behaviours”**	-	-	-	-	-	-	0.00 (0.00)	1.00 (0.99–1.00)	-	-	-	-	-	-
**EBI parental bond**	**ECAP class “allergen-avoidance”**	-	-	-	-	-	-	-	-	0.09 (0.09)	1.10 (0.93–1.29)	-	-	-	-
	**ECAP class “mixed behaviours”**	-	-	-	-	-	-	-	-	0.17 (0.10)	1.19 (0.98–1.45)	-	-	-	-
HL*EBI parental bond	**ECAP class “allergen-avoidance”**	-	-	-	-	-	-	-	-	0.00 (0.00)	1.00 (0.99–1.00)	-	-	-	-
	**ECAP class “mixed behaviours”**	-	-	-	-	-	-	-	-	-0.01 (0.00)	1.00 (0.99–1.00)	-	-	-	-
**PHQ mild depression**	**ECAP class “allergen-avoidance”**	-	-	-	-	-	-	-	-	-	-	1.09 (0.64)	2.98 (0.85–10.44)	-	-
	**ECAP class “mixed behaviours”**	-	-	-	-	-	-	-	-	-	-	0.79 (0.77)	2.21 (0.49–10.01)	-	-
**PHQ major depression**	**ECAP class “allergen-avoidance”**	-	-	-	-	-	-	-	-	-	-	0.83 (1.02)	2.30 (0.31–16.96)	-	-
	**ECAP class “mixed behaviours”**	-	-	-	-	-	-	-	-	-	-	2.11 (1.11)	8.23 (0.93–72.77)	-	-
HL*PHQ mild depression	**ECAP class “allergen-avoidance”**	-	-	-	-	-	-	-	-	-	-	-0.03 (0.02)	0.97 (0.93–1.00)	-	-
	**ECAP class “mixed behaviours”**	-	-	-	-	-	-	-	-	-	-	-0.02 (0.02)	0.98 (0.94–1.02)	-	-
HL*PHQ major depression	**ECAP class “allergen-avoidance”**	-	-	-	-	-	-	-	-	-	-	-0.02 (0.03)	0.98 (0.92–1.04)	-	-
	**ECAP class “mixed behaviours”**	-	-	-	-	-	-	-	-	-	-	-0.05 (0.03)	0.96 (0.90–1.02)	-	-
**PHQ anxiety**	**ECAP class “allergen-avoidance”**	-	-	-	-	-	-	-	-	-	-	-	-	0.00 (2.09)	1.00 (0.02–59.28)
	**ECAP class “mixed behaviours”**	-	-	-	-	-	-	-	-	-	-	-	-	0.80 (2.23)	2.22 (0.03–176.24)
HL*PHQ anxiety	**ECAP class “allergen-avoidance”**	-	-	-	-	-	-	-	-	-	-	-	-	0.01 (0.06)	1.00 (0.89–1.13)
	**ECAP class “mixed behaviours”**	-	-	-	-	-	-	-	-	-	-	-	-	-0.01 (0.06)	0.99 (0.87–1.12)

Model 0: univariable model; models 1–6: adjusted for migration background, social status and number of children. Models 2–6: including moderators (model 2 includes allergy risk, model 3 EBI parental competence, model 4 EBI parental bond, model 5 PHQ depression, and model 6 PHQ anxiety)

ECAP class 1 “breastfeeding”: *N* = 871, ECAP class 2 “allergen-avoidance”: *N* = 490, ECAP class 3 “mixed behaviours”: *N* = 301

McFadden’s pseudo-R^2^: model 1–6: 0.01 < *R*^2^ < 0.03

*HL* Health literacy score (0–50), *SE* Standard error, *OR* Odds ratio, *CI* Confidence interval, *EBI* “Eltern-Belastungs-Inventar” (parenting stress index)

As moderation analyses did not reveal significant results the final multivariable model is model 1. Model 1 was significantly different to the univariable model. The amount of explained variance was small (McFadden’s pseudo-*R*^2^ = 0.01).

## Discussion

We showed an effect of mothers’ HL on ECAP behaviours: lower HL led mothers to behaviours such as feeding hypoallergenic infant milk, avoiding specific foods in the child’s diet during the first year of life or implementing measures against house dust mites. The size of the effect was small but remained statistically significant after adjusting for potential confounding variables. None of the potentially moderating variables we tested significantly altered the effect of mothers’ HL on ECAP behaviours.

We identified different patterns of behaviours that are practiced in families in Germany in relation to ECAP. When comparing our classes to recommendations the class “breastfeeding” includes behaviours that mostly reflect current recommendations, the class “allergen-avoidance” encompasses behaviours that are discouraged in guidelines since 2014 [13, 14]. It is remarkable that a considerable proportion of mothers still avoid allergens in the diet and home environment of their child.

Our finding of an effect of HL on ECAP behaviours contributes to the large body of evidence on high parental HL and favourable health behaviours [4]. However, our goal to identify additional variables probably influencing the effect of HL on health behaviours was not achieved. Neither allergy risk status, nor mothers’ bond, competence, depression or anxiety were moderators of the effect of HL on ECAP, respectively. Thus, we can assume that HL is related to ECAP behaviours, regardless of whether a child is at-risk of allergy or not at-risk, whether mothers have high or low parental competence, strong or weak child bonding or present symptoms of mental disorders.

Our study makes a unique contribution to the field of HL research in childhood allergy prevention. We did not restrict our analyses to single behaviours, but we operationalized ECAP as a broad set of several specific health behaviours that might be related to allergy prevention, regardless of whether they are currently recommended or not. This comprehensive perspective acknowledges that ECAP touches many different areas of child care, in contrast to previous studies that focused mostly on associations between HL and specific allergy prevention related behaviours [19–21].

Especially health professionals involved in the counselling of families during pregnancy or the first months could support parents in accessing, understanding, appraising and applying information about child health and ECAP, respectively. However, HL-sensitive and HL-supportive counselling techniques are only poorly used which often leads to a systematic overestimation of parental HL and, as a result, a lack of knowledge among parents [36, 37]. Subsequently, parents often base health-related decisions on other sources like asking peers or intuition [38]. Developing comprehensible evidence-based ECAP information and providing easy access for families might be helpful. In addition, there is further potential to strengthen parents’ HL through specific interventions. Until now, HL interventions are still underutilized, though recent studies already addressed different HL dimensions like enhancing access and utilization of health information or support services. Particularly, home- and community-based programs as well as interventions delivered by midwives, public health nurses, community-health workers and trained peer mothers played a crucial role in developing trust, social support and overcoming barriers in accessing and utilizing health care and support services [39].

### Strengths and limitations

The analyses of this study as well as the selection of moderating and confounding variables were based on previous literature, a conceptual model of HL [1], and DAGs methodology. A statistical analysis plan was registered a priori [32]. Mothers’ HL was assessed using the health care scale of the HLS-EU-Q47, a common validated standardized questionnaire. For feasability reasons only a subscale with 16 items was used. It consists of self-ratings about how easy or difficult people perceive their dealing with health information. Possibly social desirability or misjudgement of competences could have biased the results. Additionally, data collection within the KUNO-Kids health study is extensive causing missing values. For missing values that were detected in the analysis sample we applied multiple imputation in order to use all available information without the need of listwise deletion of participants with missing values in single variables. Further, selection bias through drop-out is a common problem in cohort studies. Participants who dropped out during the first year of the study were characterized by lower sociodemographic levels. Despite the use of statistical imputation methods, it is important to interpret the results with caution and recognize that they may not be applicable to mothers with different sociodemographic characteristics. While it is a strength of our study that we adopted a comprehensive perspective on ECAP, this approach also entails limitations. Breastfeeding, which was the most prominent variable in the pattern of class 1, is generally important for the health of both mother and child and is part of various child health recommendations that do not specifically focus on ECAP [40, 41]. As a result, it is unclear whether mothers assigned to class 1 “breastfeeding” are intentionally engaging in ECAP behaviours or are more likely to engage in general health behaviours. Therefore, further research is needed to understand when and how parental HL translates into health behaviours or ECAP. Additionally, future studies should consider other variables that might contribute to the effect of HL on ECAP, as the amount of explained variance in our models was quite small. Due to the availability of variables in the study, not all potentially relevant variables based on recent literature and theoretical considerations could be included in the DAG. We acknowledge that this does not reflect a perfect DAG with all possible variables and pathways, and further research is needed to address this limitation.

## Conclusions

Mothers’ HL had an effect on ECAP behaviours. Improving HL could contribute to the implementation of recommended ECAP behaviours in families, especially to the reduction of allergen-avoiding behaviours. However, further research on underlying relations is needed as the included moderators in our analyses did not contribute to the understanding of the mechanisms between HL and health behaviours.

## Supplementary Information

Supplementary Material 1.

Supplementary Material 2.

Supplementary Material 3.

Supplementary Material 4.

## Data Availability

Data analysed for this study can be obtained from the corresponding author on reasonable request.
