# Supplementary material for: Effect of mothers‘ health literacy on early childhood allergy prevention behaviours: results from the KUNO-Kids health study
Source: BMC Public Health. 2024 Sep 5;24:2420. doi: 10.1186/s12889-024-19906-8 (PMC11375835; doi:10.1186/s12889-024-19906-8)
Supplement: Supplementary file 1 — Supplementary Material 1. [file 12889_2024_19906_MOESM1_ESM.docx]

Supplementary file I: Constructs/variables and questionnaire items (translated into English)

| Construct/variable (time points for data collection) | Questionnaire items |
| --- | --- |
| Outcome variable (ECAP) |  |
| Fish as part of mothers’ diet during pregnancy (baseline) | Which of the following foods did you eat during your pregnancy? (Fish)   - Once a month - Several times a month - About once a week - Several times a week - Almost daily - Never |
| Duration of exclusively breastfeeding (6 months) | How long was your child exclusively breastfed without complementary foods?   - [free text entry months, weeks, days] |
| Regular feeding of hydrolysed infant formula (6 months) | Please state the product name and company of the infant milk or infant milk substitute.   - [free text entry] |
| Child’s age of introduction of any solid foods (6 months) | Have you fed your child complementary food so far? If so, from what age and how often? (cow milk, vegetable mash, fruit mash, cereal porridge, cheese, fish, meat, health-promoting dairy products, hen egg, peanut, honey, sugar, other solid foods)   - [free text entry month] |
| Allergy prevention related avoidance of feeding specific foods in the child’s diet during the first year of life (6 months, one year) | Are there certain foods that you have consciously avoided in your child's diet?   - Yes - No   Which foods were these?   - Dairies - Wheat - hen egg - fish - meat - nuts (incl. peanuts) - soy - citruses - other fruit or vegetable - other foods   Why did you avoid these foods in your child's diet?   - Due to allergies or respiratory problems of the child - For the prevention of allergies - Because it was recommended by a doctor - For another reason |
| Fish as part of solid foods during the child’s first year of life (one year) | What complementary food has your child received since the last survey and how often? (fish)   - Yes - No - If yes, from which month - Once a month - Several times a month - Once a week - Several times a week - Almost daily - Several times a day |
| Feeding of farm milk (6 months, one year) | Have you fed your child any of the following types of milk directly from your own or another farm?   - cow milk not boiled - cow milk boiled - goat milk - No |
| Measures for reducing house dust mites (6 months, one year) | Have you taken any measures to prevent allergies?   - Yes, due to allergies or respiratory problems of the child - Yes, due to allergies or respiratory problems of other family members - Yes, for the prevention of allergies - No   If yes, which of the following measures have you taken?   - Carpeting removed or not purchased - Vacuum cleaner with fine dust filter or microfilter - Pillow and/or blanket with foam or synthetic filling purchased for the child - Purchased an allergy mattress for the child - Additional cleaning work or more frequent than normal washing or boiling of bed linen - Avoid contact with the animal or staying in the stable - Quit smoking in the home or cut down on smoking - Mattresses covered with mite-proof covers - Other [free text entry] |
| Exposure to tobacco smoke by smoking of parents or in the child’s home (4 weeks, 6 months, one year) | Have you smoked cigarettes regularly since the birth of your child?   - Yes - No   Have you regularly consumed alternative smoking products since the birth of your child?   - Yes - No   Have you smoked in your home since your child was born?   - Yes - No   Do you currently smoke cigarettes regularly?   - Yes - No   How many cigarettes are smoked by other people in your home on average per day?   - [free text entry] |
| Exposure variable | |
| Health literacy (baseline) | *HLS-EU-Q47 health care scale (Sørensen et al., 2013)* |
| Confounding and moderating variables | |
| Allergy risk status (baseline, 4 weeks, 6 months, one year) | *Sibling:*  Does or did this sibling have one of the following allergologic, pneumologic, skin or autoimmune diseases diagnosed by a physician? (Allergic rhinitis, allergic conjunctivitis, asthma bronchiale, neurodermatitis)   - Yes - No   *Mother:*  Do you have or have you ever had any of the following conditions diagnosed by a doctor? (Allergic rhinitis/allergic conjunctivitis, asthma bronchiale, neurodermatitis)   - Yes - No   Have you had the following complaints or illnesses since the birth of your child? (Hay fever/allergic conjunctivitis, neurodermatitis/atopic dermatitis, food allergy)   - Yes - No   Have you had the following complaints or illnesses in the last six months since the birth of your child? (Hay fever/allergic conjunctivitis, neurodermatitis/atopic dermatitis, food allergy)   - Yes - No   Have you developed the following complaints or illnesses since the last survey? (Hay fever/allergic conjunctivitis, neurodermatitis/atopic dermatitis, food allergy)   - Yes - No   *Father*:  Do you have or have you had one or more of the following allergologic and pneumologic clinical pictures, as well as skin and autoimmune diseases? (Allergic rhinitis, allergic conjunctivitis, asthma bronchiale, neurodermatitis)   - Yes - No   Have you developed the following complaints or illnesses since the last survey? (Hay fever/allergic conjunctivitis, neurodermatitis/atopic dermatitis, food allergy)   - Yes - No |
| EBI parental competence scale & bonding scale  (4 weeks) | *Tröster, 2010* |
| PHQ-D depression scale & anxiety scale (4 weeks) | *Löwe et al., 2002* |
| Migration background (baseline) | In which country were you born? Please use the current country name.   - [free text entry] |
| Subjective social status (4 weeks) | *MacArthur Scale (Hoebel et al., 2015)* |
| Number of children (baseline) | Is this your first child?   - Yes - No |
| Sociodemographic variables |  |
| Mothers’s age (baseline) | *Clinical routine data* |
| Marital status (baseline) | What is your marital status?   - Married - Married, living separately from spouse - Single, but living with a steady partner - Single, without a steady partner - Divorced - Widowed |
| Maternal education (baseline) | What is your highest general school-leaving qualification?   - Pupil, attending a full-time general education school - Left school without a secondary school leaving certificate (Volksschulabschluss) - Secondary school leaving certificate (Volksschulabschluss) - Secondary school leaving certificate (Mittlere Reife) - GDR polytechnic secondary school with completion of 8th, 9th or 10th grade - Fachhochschulreife, graduation from a Fachoberschule - General or subject-specific higher education entrance qualification/Abitur (Gymnasium or EOS, also EOS apprenticeship) - A-levels completed via second-chance education - Another school-leaving qualification |
| Maternal employment before pregnancy (baseline) | Were you gainfully employed before you went on maternity leave, i.e. were you in paid employment?   - Yes - No |
