# Supplementary material for: Effect of mothers‘ health literacy on early childhood allergy prevention behaviours: results from the KUNO-Kids health study
Source: BMC Public Health. 2024 Sep 5;24:2420. doi: 10.1186/s12889-024-19906-8 (PMC11375835; doi:10.1186/s12889-024-19906-8)
Supplement: Supplementary file 2 — Supplementary Material 2. [file 12889_2024_19906_MOESM2_ESM.docx]

Supplementary file II: Model selection for LCA including fit indices (final model indicated in bold)

| \| Fit for 1 latent classes: \| \| --- \| \| ============================================== \| \| number of observations: 1662 \| \| number of estimated parameters: 12 \| \| residual degrees of freedom: 1650 \| \| maximum log-likelihood: -8946.85 \| \|  \| \| AIC(1): 17917.7 \| \| BIC(1): 17982.69 \| \| G^2(1): 1957.894 (Likelihood ratio/deviance statistic) \| \| X^2(1): 7118.589 (Chi-square goodness of fit) \| \| Entropy: - \|   Estimated class population shares: 100% | \| Fit for 2 latent classes: \| \| --- \| \| ============================================== \| \| number of observations: 1662 \| \| number of estimated parameters: 25 \| \| residual degrees of freedom: 1637 \| \| maximum log-likelihood: -8469.322 \| \|  \| \| AIC(2): 16988.64 \| \| BIC(2): 17124.04 \| \| G^2(2): 1002.839 (Likelihood ratio/deviance statistic) \| \| X^2(2): 2780.685 (Chi-square goodness of fit) \| \| Entropy: 0.781 \| \| LMRT: LR = 955.055, LMR LR (df = 13) = 913.973, p<0.001 \|   Estimated class population shares: 44.21%, 55.79% |
| --- | --- | --- | --- | --- | --- | --- | --- | --- | --- | --- | --- | --- | --- | --- | --- | --- | --- | --- | --- | --- | --- | --- | --- | --- | --- | --- |
| \| **Fit for 3 latent classes:** \| \| --- \| \| **==============================================** \| \| **number of observations: 1662** \| \| **number of estimated parameters: 38** \| \| **residual degrees of freedom: 1624** \| \| **maximum log-likelihood: -8343.003** \| \|  \| \| **AIC(3): 16762.01** \| \| **BIC(3): 16967.8** \| \| **G^2(3): 750.2003 (Likelihood ratio/deviance statistic)** \| \| **X^2(3): 2350.085 (Chi-square goodness of fit)** \| \| **Entropy: 0.729** \| \| **LMRT: LR = 252.639, LMR LR (df = 13) = 241.772, p<0.001** \|   **Estimated class population shares: 53.91%, 27.52%, 18.57%** | \| Fit for 4 latent classes: \| \| --- \| \| ============================================== \| \| number of observations: 1662 \| \| number of estimated parameters: 51 \| \| residual degrees of freedom: 1611 \| \| maximum log-likelihood: -8303.68 \| \|  \| \| AIC(4): 16709.36 \| \| BIC(4): 16985.56 \| \| G^2(4): 671.5552 (Likelihood ratio/deviance statistic) \| \| X^2(4): 2264.613 (Chi-square goodness of fit) \| \| Entropy: 0.521 \| \| LMRT: LR = 78.645, LMR LR (df = 13) = 75.262, p<0.001 \|   Estimated class population shares: 35.94%, 17.88%, 19.32%, 26.86% |
| \| Fit for 5 latent classes: \| \| --- \| \| ============================================ \| \| number of observations: 1662 \| \| number of estimated parameters: 64 \| \| residual degrees of freedom: 1598 \| \| maximum log-likelihood: -8275.561 \| \|  \| \| AIC(5): 16679.12 \| \| BIC(5): 17025.73 \| \| G^2(5): 615.3169 (Likelihood ratio/deviance statistic) \| \| X^2(5): 2300.086 (Chi-square goodness of fit) \| \| Entropy: 0.379 \| \| LMRT: LR = 56.238, LMR LR (df = 13) = 53.819, p<0.001 \|   Estimated class population shares: 32.95%  17.27%, 5.65%, 26.69%, 17.43% | \| Fit for 6 latent classes: \| \| --- \| \| ============================================== \| \| number of observations: 1662 \| \| number of estimated parameters: 77 \| \| residual degrees of freedom: 1585 \| \| maximum log-likelihood: -8253.957 \| \|  \| \| AIC(6): 16661.91 \| \| BIC(6): 17078.93 \| \| G^2(6): 572.1082 (Likelihood ratio/deviance statistic) \| \| X^2(6): 2076.207 (Chi-square goodness of fit) \| \| Entropy: 0.37 \| \| LMRT: LR = 43.209, LMR LR (df = 13) = 41.350, p<0.001 \|   Estimated class population shares: 17.34%, 20.13%, 7.96%, 33.27%, 5.94%, 15.35% |
