# Supplementary material for: Effect of mothers‘ health literacy on early childhood allergy prevention behaviours: results from the KUNO-Kids health study
Source: BMC Public Health. 2024 Sep 5;24:2420. doi: 10.1186/s12889-024-19906-8 (PMC11375835; doi:10.1186/s12889-024-19906-8)
Supplement: Supplementary file 3 — Supplementary Material 3. [file 12889_2024_19906_MOESM3_ESM.docx]

Supplementary file III: STROBE Statement: checklist for cohort studies

|  | Item No | | | Recommendation | Page No |  |
| --- | --- | --- | --- | --- | --- | --- |
| **Title and abstract** | 1 | | | (*a*) Indicate the study’s design with a commonly used term in the title or the abstract | 1 | |
|  |  |  |  | (*b*) Provide in the abstract an informative and balanced summary of what was done and what was found | 2 |  |
| Introduction | | | | | |  |
| Background/rationale | 2 | | | Explain the scientific background and rationale for the investigation being reported | 3-4 |  |
| Objectives | 3 | | | State specific objectives, including any prespecified hypotheses | 4 |  |
| Methods | | | | | |  |
| Study design | 4 | | | Present key elements of study design early in the paper | 4 |  |
| Setting | 5 | | | Describe the setting, locations, and relevant dates, including periods of recruitment, exposure, follow-up, and data collection | 4-5 |  |
| Participants | 6 | | | (*a*) Give the eligibility criteria, and the sources and methods of selection of participants. Describe methods of follow-up | 4-5 |  |
|  |  |  |  | (*b*) For matched studies, give matching criteria and number of exposed and unexposed |  |  |
| Variables | 7 | | | Clearly define all outcomes, exposures, predictors, potential confounders, and effect modifiers. Give diagnostic criteria, if applicable | 5-7, Figure 1, Supplement I |  |
| Data sources/ measurement | 8* | | | For each variable of interest, give sources of data and details of methods of assessment (measurement). Describe comparability of assessment methods if there is more than one group | 5-7, Supplement I |  |
| Bias | 9 | | | Describe any efforts to address potential sources of bias | 7 |  |
| Study size | 10 | | | Explain how the study size was arrived at | 5 |  |
| Quantitative variables | 11 | | | Explain how quantitative variables were handled in the analyses. If applicable, describe which groupings were chosen and why | 7-8 |  |
| Statistical methods | 12 | | | (*a*) Describe all statistical methods, including those used to control for confounding | 7-8 |  |
|  |  |  |  | (*b*) Describe any methods used to examine subgroups and interactions | 7-8 |  |
|  |  |  |  | (*c*) Explain how missing data were addressed | 7 |  |
|  |  |  |  | (*d*) If applicable, explain how loss to follow-up was addressed | 7 |  |
|  |  |  |  | (*e*) Describe any sensitivity analyses |  |  |
| Results | | | | |  |  |
| Participants | 13* | | | (*a*) Report numbers of individuals at each stage of study—eg numbers potentially eligible, examined for eligibility, confirmed eligible, included in the study, completing follow-up, and analysed | 8, Table 1, Supplement IV |  |
|  |  |  |  | (*b*) Give reasons for non-participation at each stage |  |  |
|  |  |  |  | (*c*) Consider use of a flow diagram |  |  |
| Descriptive data | 14* | | | (*a*) Give characteristics of study participants (eg demographic, clinical, social) and information on exposures and potential confounders | 8, Table 1 |  |
|  |  |  |  | (*b*) Indicate number of participants with missing data for each variable of interest |  |  |
|  |  |  |  | (*c*) Summarise follow-up time (eg, average and total amount) |  |  |
| Outcome data | 15* | | | Report numbers of outcome events or summary measures over time | 9 |  |
| Main results | | 16 | (*a*) Give unadjusted estimates and, if applicable, confounder-adjusted estimates and their precision (eg, 95% confidence interval). Make clear which confounders were adjusted for and why they were included | | 9-11, Figure 2, Table 2 |  |
|  |  |  | (*b*) Report category boundaries when continuous variables were categorized | | 8 |  |
|  |  |  | (*c*) If relevant, consider translating estimates of relative risk into absolute risk for a meaningful time period | |  |  |
| Other analyses | | 17 | Report other analyses done—eg analyses of subgroups and interactions, and sensitivity analyses | | Supplement V |  |
| Discussion | | | | | |  |
| Key results | | 18 | Summarise key results with reference to study objectives | | 14-15 |  |
| Limitations | | 19 | Discuss limitations of the study, taking into account sources of potential bias or imprecision. Discuss both direction and magnitude of any potential bias | | 15-16 |  |
| Interpretation | | 20 | Give a cautious overall interpretation of results considering objectives, limitations, multiplicity of analyses, results from similar studies, and other relevant evidence | | 14-15 |  |
| Generalisability | | 21 | Discuss the generalisability (external validity) of the study results | | 15 |  |
| Other information | | | | | |  |
| Funding | | 22 | Give the source of funding and the role of the funders for the present study and, if applicable, for the original study on which the present article is based | | 16 |  |
