# Supplementary material for: Effect of mothers‘ health literacy on early childhood allergy prevention behaviours: results from the KUNO-Kids health study
Source: BMC Public Health. 2024 Sep 5;24:2420. doi: 10.1186/s12889-024-19906-8 (PMC11375835; doi:10.1186/s12889-024-19906-8)
Supplement: Supplementary file 4 — Supplementary Material 4. [file 12889_2024_19906_MOESM4_ESM.docx]

Supplementary file IV: General characteristics at baseline of participants who were included (analysis sample) and not included (drop-out sample), total KUNO-Kids sample N=3199

|  | $\text{N}^{\text{a}}$ (analysis sample, $\text{N}^{\text{a}}$=1662) | | | $\text{N}^{\mathbf{b}}$ (drop-out sample, $\text{N}^{\mathbf{b}}$=1537) | | | |  |  |
| --- | --- | --- | --- | --- | --- | --- | --- | --- | --- |
|  | **N** | **N (%)** | **Mean (SD)** |  | **N** | **N (%)** | **Mean (SD)** | **p** | |
| Age (years) | 1647 |  | 32.61 (4.10) |  | 1518 |  | 31.78 (4.73) | <.001 | |
| Marital status | 1629 |  |  |  | 1483 |  |  | <.001 | |
| Married,  living  together  with  husband |  | 1333 (81.8%) |  |  |  | 1113 (75.1%) |  |  | |
| Unmarried,  living  together  with  partner |  | 271 (16.6%) |  |  |  | 319 (21.5%) |  |  | |
| Living  without  partner/  divorced/  widowed |  | 25 (1.5%) |  |  |  | 51 (3.4%) |  |  | |
| Migration background (country of birth Germany) | 1631 | 1482 (90.9%) |  |  | 1485 | 1199 (80.7%) |  | <.001 | |
| Maternal education | 1626 |  |  |  | 1471 |  |  | <.001 | |
| No degree or  less than 10  years of  schooling |  | 107 (6.6%) |  |  |  | 218 (14.8%) |  |  | |
| Ten years of  schooling |  | 494 (30.4%) |  |  |  | 498 (33.9%) |  |  | |
| University  entrance  level |  | 1025 (63.0%) |  |  |  | 755 (51.3%) |  |  | |
| Maternal employment before pregnancy | 1627 | 1481 (91.0%) |  |  | 1482 | 1265 (85.4%) |  | <.001 | |
| Primiparous | 1644 | 1048 (63.7%) |  |  | 1499 | 799 (53.3%) |  | <.001 | |

$\text{N}^{\text{a}}$: analysis sample; $\text{N}^{b}$: drop-out sample; SD: standard deviation
